# Changes in rat intestinal motility and absorption following intestinal transplantation: an experimental study

**DOI:** 10.3389/ti.2026.17141

**Published:** 2026-10-09

**Authors:** Antoine Dubois, Amélie Leenders, Gert De Hertogh, Alice Rustichelli, Caroline Boelhouwer, Tine Wylin, Veerle Heedfeld, Eliano Bonaccorsi-Riani, Jacques Pirenne, Tim Vanuytsel, Ricard Farré, Laurens J. Ceulemans

**Affiliations:** 1 Leuven Intestinal Failure and Transplantation (LIFT), UZ Leuven, Leuven, Belgium; 2 Laboratory of Abdominal Transplantation, Department of Microbiology, Immunology and Transplantation, KU Leuven, Leuven, Belgium; 3 Pôle de Chirurgie Expérimentale et Transplantation, UC Louvain, Brussels, Belgium; 4 Translational Cell and Tissue Research, Department of Imaging and Pathology, KU Leuven, Leuven, Belgium; 5 Department of Chronic Diseases and Metabolism (CHROMETA), Translation Research Center for Gastrointestinal Disorders (TARGID), KU Leuven, Leuven, Belgium; 6 Laboratory of Respiratory Diseases and Thoracic Surgery (BREATHE), Department of Chronic Diseases and Metabolism (CHROMETA), KU Leuven, Leuven, Belgium

**Keywords:** graft function, histology, intestinal transplantation, intestine, permeability

## Abstract

Intestinal transplantation (ITx) is a life-saving procedure for patients with complicated intestinal failure. In the acute phase, allograft permeability increases primarily due to ischemia-reperfusion injury. However, long-term allograft physiology after transplantation remains poorly understood, particularly the impact of lost autonomous graft innervation. This study aims to evaluate post-ITx allograft motility and permeability using orally administered fluorescein-labeled dextrans (TRITC70,FITC4). Lewis-to-Lewis orthotopic ITx (n = 6, 260–300 g) were performed. Intestinal permability and motility were evaluated in recipient at baseline (2 days pre-transplant, control) and 4 weeks post-transplant (experimental), using orally administered dextrans (TRITC70 and FITC4). Total GI motility was assessed by TRITC70 fecal excretion, while intestinal and colonic permeability were evaluated using TRITC70 (transcellular route) and FITC4 (paracellular route) plasma concentrations, measured at 3 h, 6 h, 24 h. Paired data were reported as mean (±SEM) and compared using paired t-test (normal distribution)/Wilcoxon rank test (non-normal distribution). Fecal excretion of TRITC70 was 9.58 (±0.85) hours in the control group and 11.02 (±1.40) hours in the experimental group (p = 0.287). Plasma concentrations (µg/mL) of FITC4 at 3 h, 6h, and 24 h were (control vs*.* experimental): 0.23 (±0.08) vs*.* 0.72 (±0.23) (p = 0.041), 0.12 (±0.06) vs*.* 0.53 (±0.16) (p = 0.031), and 0.04 (±0.02) vs*.* 0.12 (±0.04) (p = 0.625). For TRITC70, plasma concentrations were 11.20 (±1.81) vs*.* 11.11 (±1.39) (p = 0.975), 20.49 (±8.87) vs*.* 12.60 (±1.87) (p = 0.562), and 20.82 (±7.25) vs*.* 5.02 (±0.82) (p = 0.094). Finally, the AUC (µgh/mL) of FITC4 and TRITC70 absorption were 2.39 (±1.1) vs*.* 8.84 (±2.69) (p = 0.029) and 436 (±159) vs*.* 197 (±25) (p = 0.195), respectively. Four weeks after ITx, we observed an increased paracellular permeability compared to pre-transplant evaluation, while overall GI motility remained unchanged. Further research is needed to better understand intestinal graft physiology.

## Graphical Abstract

**Figure:**
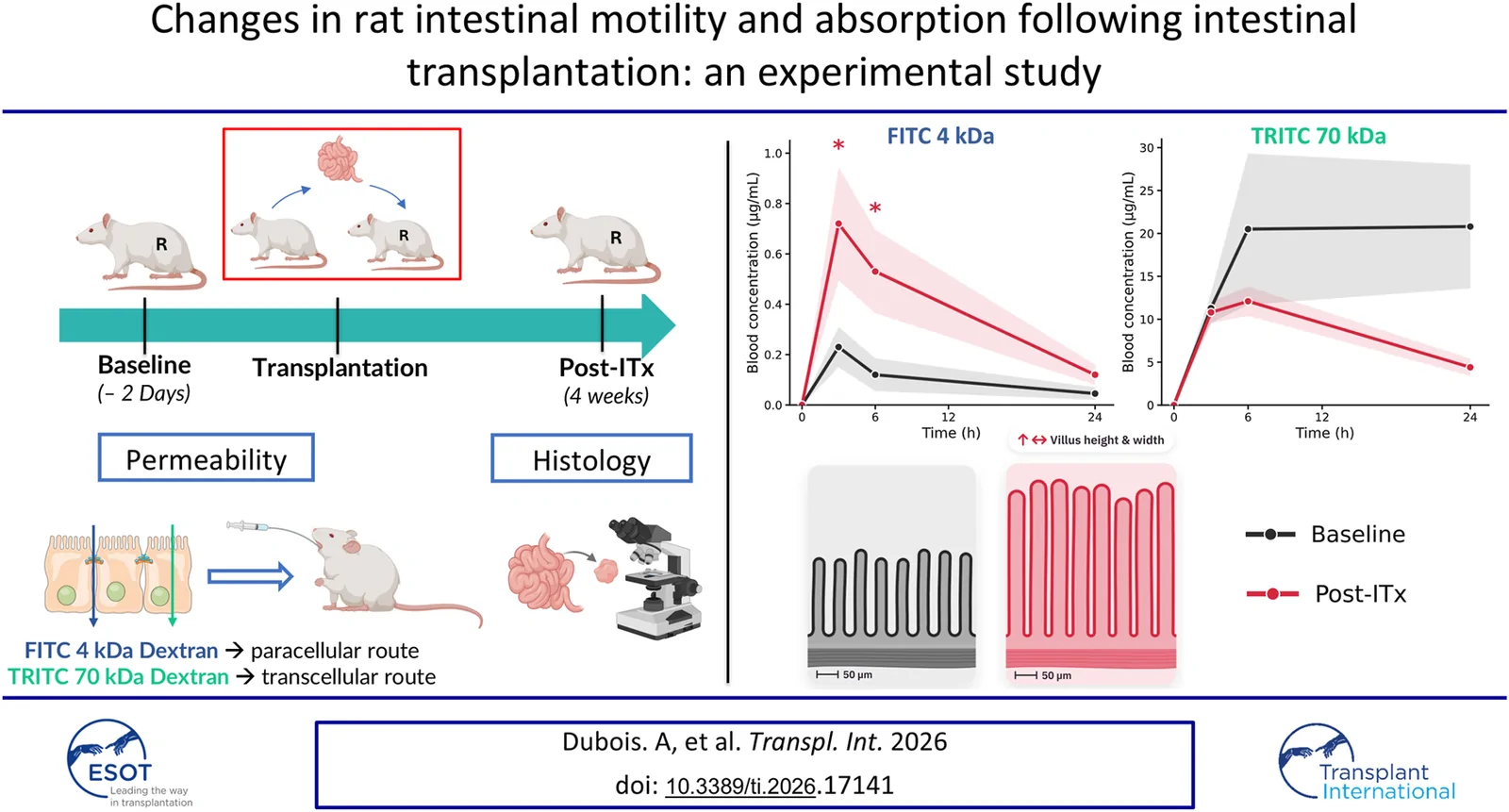

## Introduction

Intestinal transplantation (ITx) is a life-saving procedure for patients with complicated intestinal failure. Despite improvements in donor selection and post-transplant management, outcomes lag behind those of other solid organ transplants. ITx recipients frequently suffer from persistent enteric dysfunction, for which no effective targeted therapy currently exists, while the underlying mechanisms remain insufficiently understood [1–4].

During the transplantation process, graft physiology is severely altered. Procurement requires transection of lymphatic vessels, raising concerns regarding post-ITx lipid absorption. Likewise, autonomic (sympathetic and parasympathetic) innervation is disrupted, leaving the graft reliant on hormonal regulation and intrinsic enteric nervous system activity (intramuscular and submucosal ganglia) without central control [5]. Moreover, long-term functional consequences of ischemia–reperfusion injury (IRI) on the transplanted graft remain unknown and might not be trivial considering the intestine’s high sensitivity to ischemia [5, 6]. These long-term physiological and histological modifications may impair ITx outcomes and remain poorly characterized [7].

Fluorescent dextrans are non-digestible fluorophore-labeled polysaccharides that enable quantitative assessment of intestinal permeability by measuring blood fluorescence following oral administration [8, 9]. Dextran type, defined by their molecular weight and the conjugated fluorescein, allows an independent evaluation of paracellular and transcellular transport pathways [8, 10, 11]. In addition, dextran fecal excretion provides a functional measure of gastrointestinal (GI) motility.

In this pilot study, we aimed to characterize long-term functional and morphological changes associated with intestinal transplantation in a syngeneic rat ITx model using fluorescent dextrans, following recovery from the acute post-transplant phase.

## Materials and methods

### General

Six-week-old male inbred Lewis rats (N = 26) (Janvier Labs, Saint-Berthevin, France) weighing 250–350 g were used. All procedures were approved by the KU Leuven Institutional Animal Care and Use Committee (CHROMETA-144/2023) and conducted in accordance with European Union guidelines for animal welfare. Rats were housed 2–3 per cage in a dedicated facility under controlled temperature and a 14/10-h light/dark cycle, with *ad libitum* access to food and water. Postoperatively, animals were monitored daily for distress using a standardized morbidity scoring system.

### Intestinal transplantation

Syngeneic Lewis-to-Lewis ITx was chosen to avoid immunologic influences on endpoints [12, 13]. During both donor and recipient procedures, rats were placed on a heating pad, and a 24G tail vein catheter was inserted for intraoperative fluid infusion (0.9% NaCl, 0.6 mL/min; Baxter). The ITx procedure has been previously described by our group and is briefly summarized [14]. Recorded surgical data included donor and recipient weights, time of donor procedure, cold ischemia time, anastomoses time and time of recipient procedure.

#### Anesthesia

Rats were not fasted before surgery. Isoflurane (1000 mg/g, Iso-Vet, Dechra, Belgium) inhalation was used for anesthesia induction and maintenance (5% and 1.5% isoflurane, respectively), carried by 1.5 L/min O2).

#### Donor procedure

Through a midline laparotomy, the subdiaphragmatic aorta and the superior mesenteric artery (SMA) were exposed. The distal ileum was transected at 1 cm from the caecum. The colon was progressively dissected from the mesenteric, thereby removing pancreatic tissue. The jejunal end of the graft was then transected, and the portal vein (PV) isolated by ligation and division of the splenic and pyloric veins. The graft was flushed with 10 mL of cold IGL-1 solution, procured, and the intestinal lumen was subsequently flushed with cold IGL-1. The graft was stored in IGL-1 solution at 0 °C–4 °C until implantation [14, 15].

#### Recipient procedure

In the recipient, the graft was transplanted on the infra-renal aorta and vena cava (VC) by two end-to-side anastomoses: 1) PV to VC and 2) SMA to aorta. Once completed, the clamps were removed, and the graft was reperfused. The native small bowel was resected by ligating the mesenteric branches with 7-0 silk, and the intestinal continuity was restored by proximal and distal end-to-end anastomoses using 7-0 non-resorbable monofilament running sutures. Finally, the abdomen was closed in two layers with 4-0 silk.

#### Post-operative course

Recipient postoperative medications include ropivacaine (Naropin 2 mg/mL, 3 mg/kg, SC, administered at abdominal closure), Ceftriaxone (Ceftriaxone Fresenius Kabi 1g, 70 mg/kg, SC, administered once after abdominal closure), and Meloxicam (Metacam 2 mg/mL, 1–2 mg/kg, SC, administered daily during the first 72 h) while rats were kept on a heating pad for 1-2 h after surgery. Animals were checked twice daily during the first 72 h and once daily thereafter.

### Euthanasia

All animals were euthanized by exsanguination under general anesthesia 4 weeks post-ITx (end of the experiment). In case of complicated post-operative course (high morbidity score, persistent pain despite painkiller, weight loss >25%), rats were prematurely sacrificed and excluded from experimental groups.

### Experimental design

Graft modifications following ITx were studied 4 weeks after ITx. Fluorescent dextrans experiment were used to assess graft permeability and motility, while structural changes were evaluated based on histological analysis (Figure 1).

**FIGURE 1:**
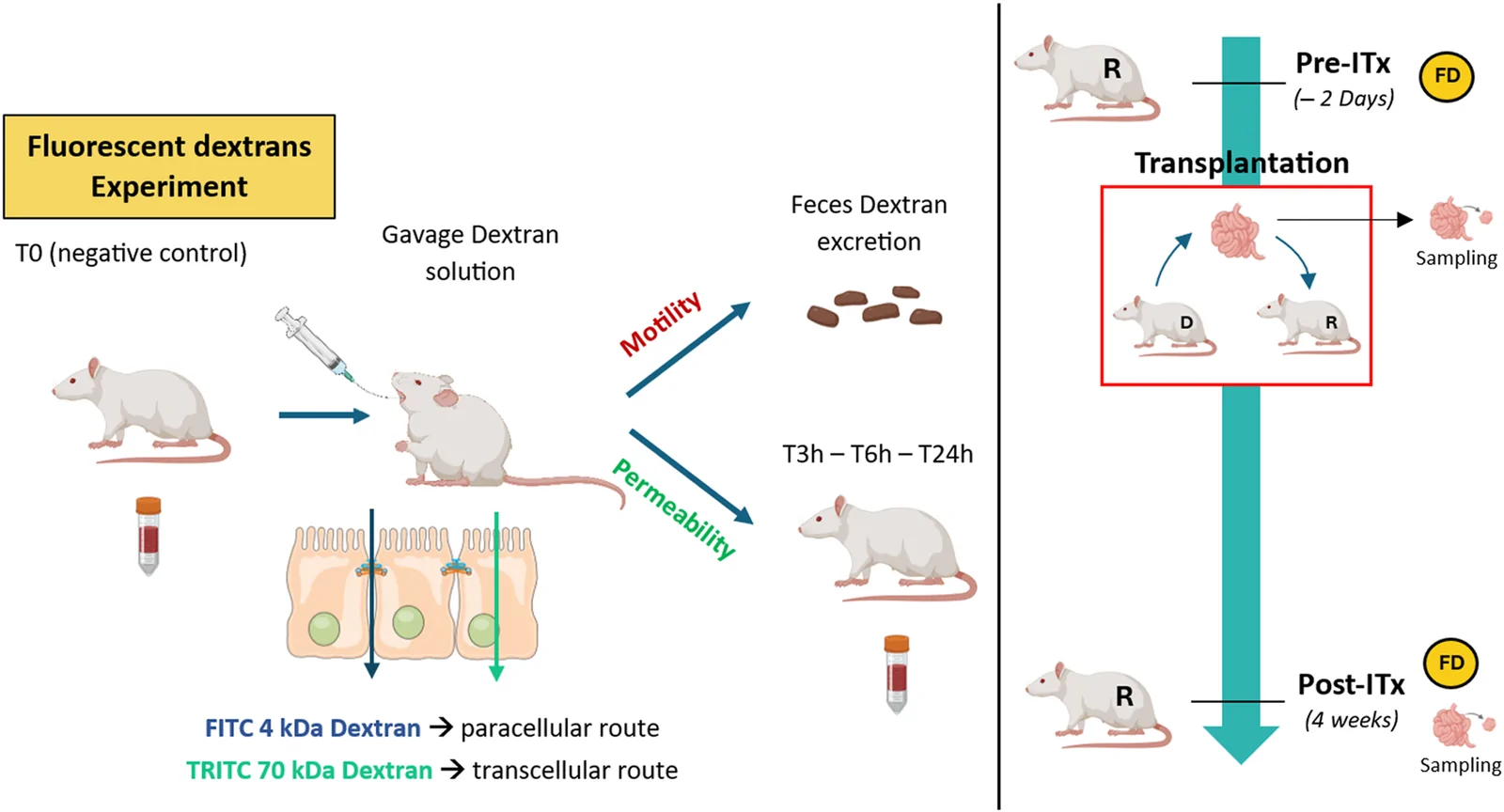
Experimental design. In the first experimental batch (n = 6), fluorescent dextrans were used to evaluate changes in intestinal permeability and motility following intestinal transplantation. After oral administration, dextrans plasma concentration at 3, 6, and 24 h was measured to assess intestinal permeability, while their excretion in feces was used to evaluate motility. Each recipient was tested both 2 days before and 4 weeks after ITx. In the second batch (n = 7), intestinal samples were collected from donor before procurement and from the transplanted grafts 4 weeks after ITx for analyses. D, donor; FD, fluorescent dextran; ITx, intestinal transplantation; R, recipient.

#### Fluorescent dextrans experiment

Measurements were performed in recipients (n = 6) 2 days pre-ITx (baseline) and repeated in the same animals 4 weeks post-ITx. Of note, experiments were performed in seven animals, but data from one rat were excluded due to technical issues. Baseline and post-ITx values were compared in a paired fashion.

Fluorescein isothiocyanate–dextran (FITC, 4 kDa) (TdB Labs, Uppsala, Sweden) was used to measure paracellular permeability considering its low molecular weight [11]. Tetramethylrhodamine isothiocyanate–dextran (TRITC, 70 kDa) (TdB Labs, Uppsala, Sweden) was used for transcellular permeability [11], while its fecal excretion (red-colored) served to monitor total GI motility. Prior to the experiment, rats were fasted for 2–3 h in separate cages with free access to water. FITC and TRITC were diluted in phosphate-buffered saline (30 mg/mL each) and administered by oral gavage (doses per dextran: 0.5 mL/100 g of body weight), under general anesthesia using similar settings as ITx. A baseline blood sample was taken by saphenous vein puncture before gavage. Animals were then allowed to recover and were re-anesthetized for sequential blood sampling at the same site at 3, 6, and 24 h after gavage. Time points were selected to distinguish the gastrointestinal site of dextran absorption: small intestine permeability at 3h, combined small intestine and colonic permeability at 6h, and late colonic permeability at 24 h.

Blood samples (200 µL) were collected in EDTA tubes and immediately centrifuged at 1200 g for 10 min at 4 °C. The supernatant was re-centrifuged at 2200 g for 15 min at 4 °C. The final supernatant was then protected from light and stored at 4 °C until analysis. Plasma was diluted 1:1 in PBS, and fluorescence was measured using a spectrophotometer (FLUOstar plate reader, BMG, Germany) in black 96-well plates (FITC: excitation 485 nm/emission 528 nm; TRITC: excitation 544 nm/emission 590 nm). Dextran concentrations were calculated using standard curves prepared in PBS (0–30 μg/mL). Fecal output was followed each hour for red-pellet excretion.

#### Histological analysis

In a second batch of experiments, histological changes were evaluated (n = 7). Terminal ileal samples were collected from donors at baseline (pre-ITx, before vascular and luminal flush) and from the graft at the time of sacrifice (4 weeks post-ITx) Tissues were formalin-fixed, paraffin-embedded, sectioned at 5 μm, and stained with hematoxylin–eosin. Assessment was performed by an experienced pathologist blinded to experimental conditions. Absorptive capacity was estimated by quantifying villus surface area and villus number per high power field (HPF; 302 μm^2^). Villus surface area (VS) was calculated by modeling each villus as a cylinder with a hemispherical tip using the lateral surface area of the cylinder (2πrh) and the curved surface area of the hemisphere (2πr^2^). Considering villus length (VL, h + r) and villus width (VW, 2r), VS was calculated according to the following formula:

$$\text{VS}=\pi \left(\text{VW}\right)\left(\text{VL}\right)\;µm^{2}$$

VL and VW were measured at four distinct locations per sample and averaged to calculate VS [16]. Numbers of villus and crypt were counted per HPF at two locations, and the crypt-to-villus ratio was calculated. Similarly, Paneth cells were counted as marker of intestinal regeneration following IRI. Finally, enteric ganglia and neurons were counted per HPF at both intramuscular and submucosal levels.

### Immunohistochemistry

Tight junctions were quantified using occludin immunohistochemistry. Formalin-fixed, paraffin-embedded sections (5 µm) were deparaffinized, rehydrated, and subjected to heat-induced antigen retrieval in citrate buffer (pH 6.0). Endogenous peroxidase was blocked with 3% H_2_O_2_, and nonspecific antibody binding was blocked using PBS-Tween20 with 5% BSA and normal goat serum (1/50). Sections were incubated overnight at 4 °C with rabbit anti-occludin antibody (Invitrogen, 40-4700; 1/100), followed by biotinylated goat anti-rabbit secondary antibody (Vector, BA-1000; 1/500) and Vectastain ABC (Vector, SK-6100). Signal was developed with DAB substrate kit (Abcam, ab64238) and counterstained with Mayer’s hematoxylin. Slides were dehydrated, mounted, and analyzed using ImageJ. Occludin-positive areas were quantified relative to hematoxylin staining using the Color Deconvolution plugin in ImageJ. Occludin and hematoxylin signals were digitally separated, and manual thresholds were applied to both channels across all images. Areas above threshold were defined as positively stained, and occludin-positive areas were normalized to hematoxylin-stained area. Analyses were performed by a blinded investigator.

### Statistics

Statistical analyses were performed using GraphPad Prism (version 9.3.1 for Windows; GraphPad Software, San Diego, CA, USA). Data are presented as scatter plots showing individual values, group means, and standard error of the mean (SEM). Data distribution was assessed using the Shapiro–Wilk test for (log)normality. Paired comparisons were performed. Normally distributed data were analyzed using a paired t-test, whereas non-normally distributed data were analyzed using the Wilcoxon matched-pairs signed-rank test. A p value <0.05 was considered statistically significant.

## Results

### Surgical data

For the 13 intestinal transplant performed, mean (min-max) donor and recipient weights were 282 (268–292) g and 281 (271–296) g, respectively. Donor procedures lasted 48 (40–58) min, followed by 61 (43–81) min of cold ischemia. Anastomoses time was 35 (27–41) min, and time for recipient procedure was 151 (133–168) min.

### Increase of the paracellular permeability after ITx

Three hours after oral gavage (intestinal permeability), FITC plasma level was significantly increased in the post-ITx group compared with baseline (p = 0.041), whereas TRITC permeability was unchanged (p = 0.975). At 6 h (mixed intestinal and colonic permeability), the FITC plasma concentration remained higher in the post-ITx group (p = 0.032), and no difference in TRITC permeability was observed (p = 0.562). At 24 h (late colonic permeability), FITC plasma concentration did not differ from baseline (p = 0.625), while TRITC showed a trend toward decreased permeability (p = 0.094). Consistently, the area under the curve (AUC) for FITC was significantly greater post-ITx than at baseline (8.84 ± 2.69 vs*.* 2.39 ± 1.10 µgh/mL; p = 0.029), whereas no statistical difference was observed for TRITC (197 ± 25 vs*.* 436 ± 159 µgh/mL; p = 0.195) (Figure 2). Occludin immunohistochemistry revealed a significant increase in relative staining area post-ITx compared to baseline samples (0.017 ± 0.002 vs*.* 0.032 ± 0.003, p = 0.002) (Figure 3). Intestinal motility, evaluated by fecal excretion of TRITC, was similar between baseline and post-ITx (p = 0.287) (Figure 4).

**FIGURE 2:**
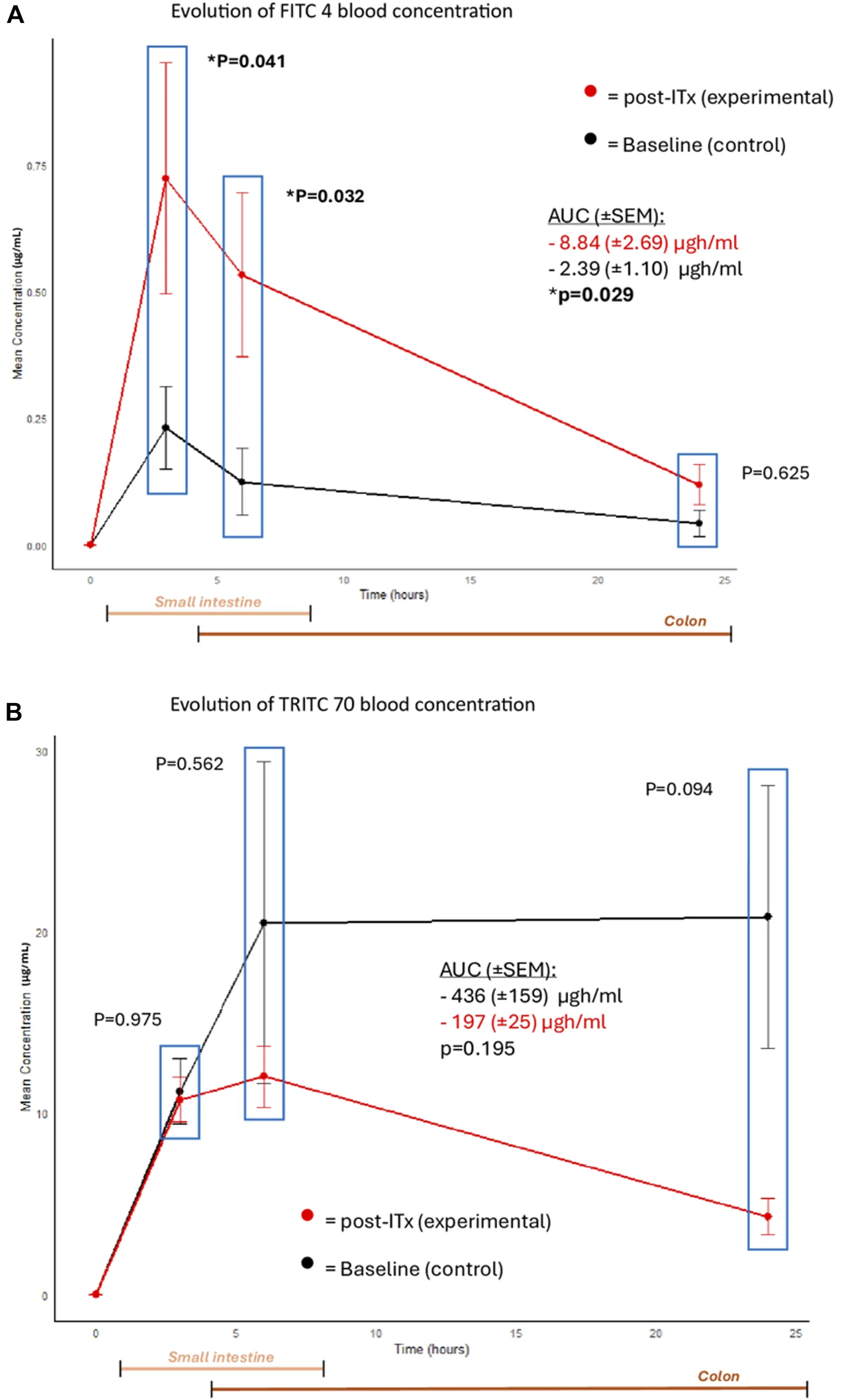
Assessment of intestinal permeability using fluorescent dextrans. Intestinal permeability of FITC4 (Panel **A**) and TRITC70 (Panel **B**) was assessed by plasma concentrations at 3, 6, and 24 h after oral gavage, reflecting small intestinal, combined small intestinal and colonic, and colonic permeability, respectively. Dextrans were administered 1 day pre intestinal transplantation (baseline) and 4 weeks post intestinal transplantation (n = 6). Paired comparisons were performed at each time point, and the area under the curve (AUC) was calculated to assess overall absorption. AUC, area under the curve; ITx, intestinal transplantation; SEM, standard error to the mean.

**FIGURE 3:**
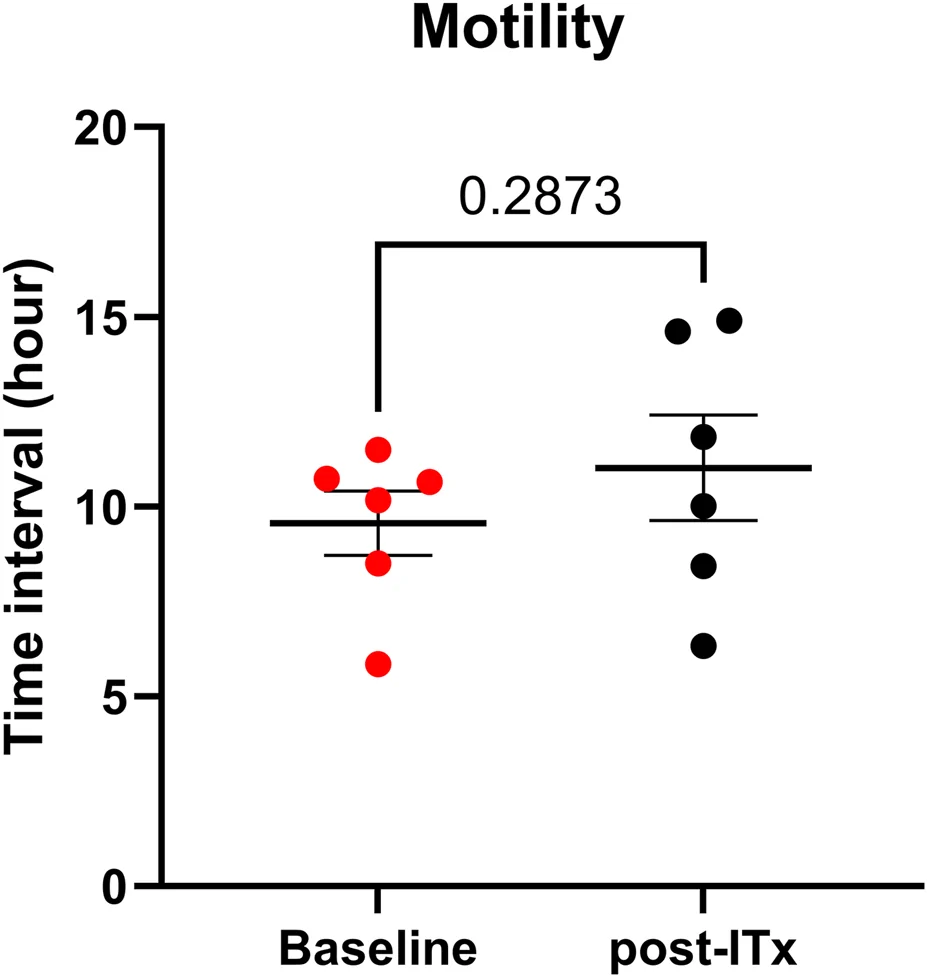
Tight junction assessment at Baseline and Post-ITx. Occludin immunohistochemistry was used to assess tight junctions pre-transplant (at procurement, baseline) and 4 weeks post-transplant (post-ITx) (n = 7). Occludin and hematoxylin signals were digitally separated, and manual thresholds were applied to both channels across all images. Areas above threshold were defined as positively stained, and occludin-positive area was normalized to hematoxylin-stained area. ITx, intestinal transplantation; VL, Villus length.

**FIGURE 4:**
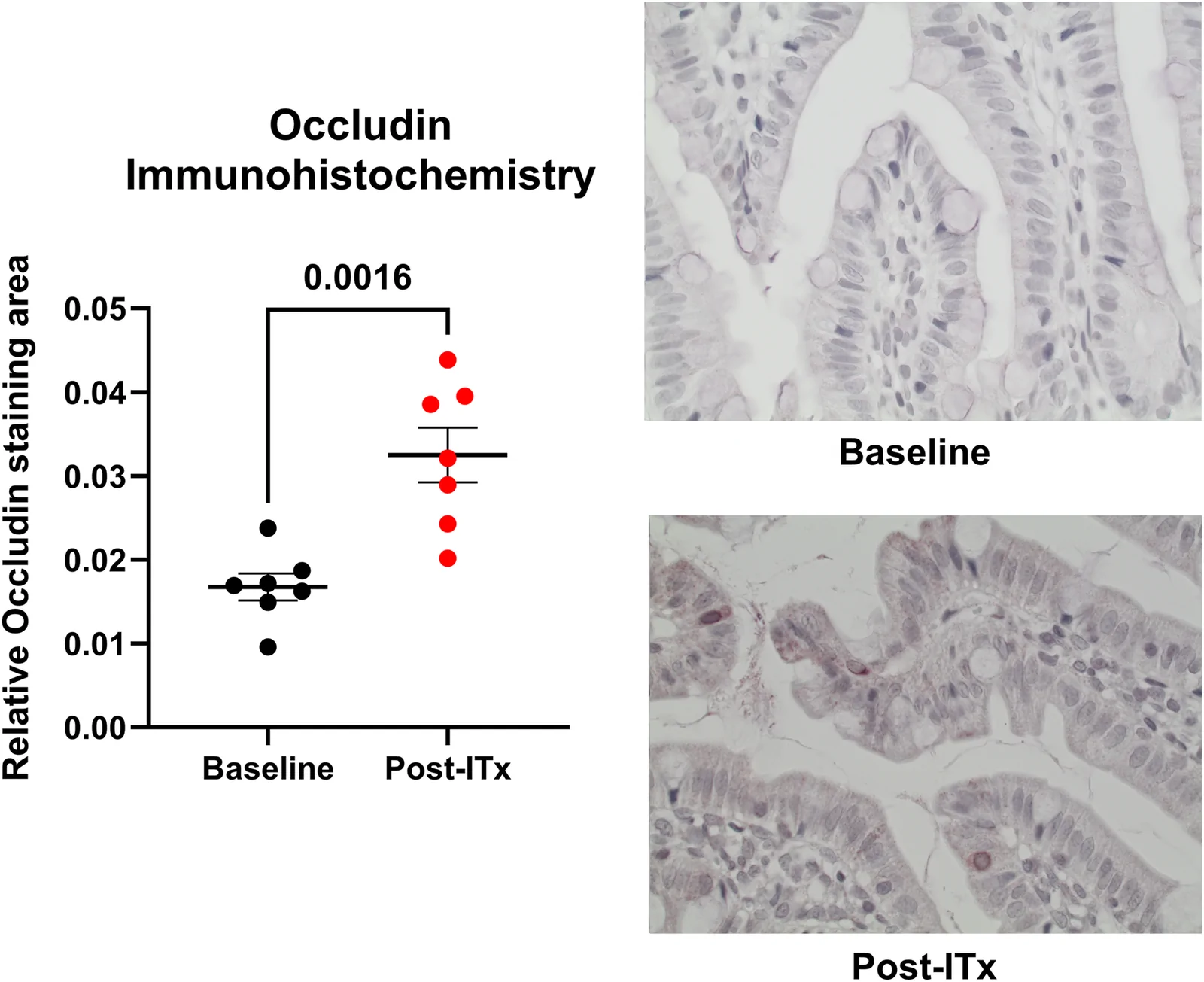
Change in intestinal motility following intestinal transplantation. Fecal excretion of TRITC70 dextran (red) was monitored following oral gavage to assess intestinal motility. The experiment was repeated in the same recipients 1 day pre-transplant (baseline) and 4 weeks post-transplant (post-ITx) (n = 6). Paired comparisons were performed. ITx, intestinal transplantation.

### Increase in villus surface area after ITx

Four weeks after ITx, histological analysis showed preserved tissue architecture without signs of injury, although VL and VW were significantly increased compared to baseline. Villus length increased from 350 ± 13 μm to 491 ± 20 µm (p = 0.016), and villus width from 79 ± 3 μm to 99 ± 4 µm (p = 0.002). Accordingly, calculated villus surface area was significantly greater post-ITx (0.153 ± 0.010 mm^2^) than at baseline (0.087 ± 0.005 mm^2^; p < 0.001). On the other hand, number of villi per HPF did not differ between baseline and post-ITx assessments (8 ± 0.43 vs. 7 ± 0.43; p = 0.359) (Figure 5).

**FIGURE 5:**
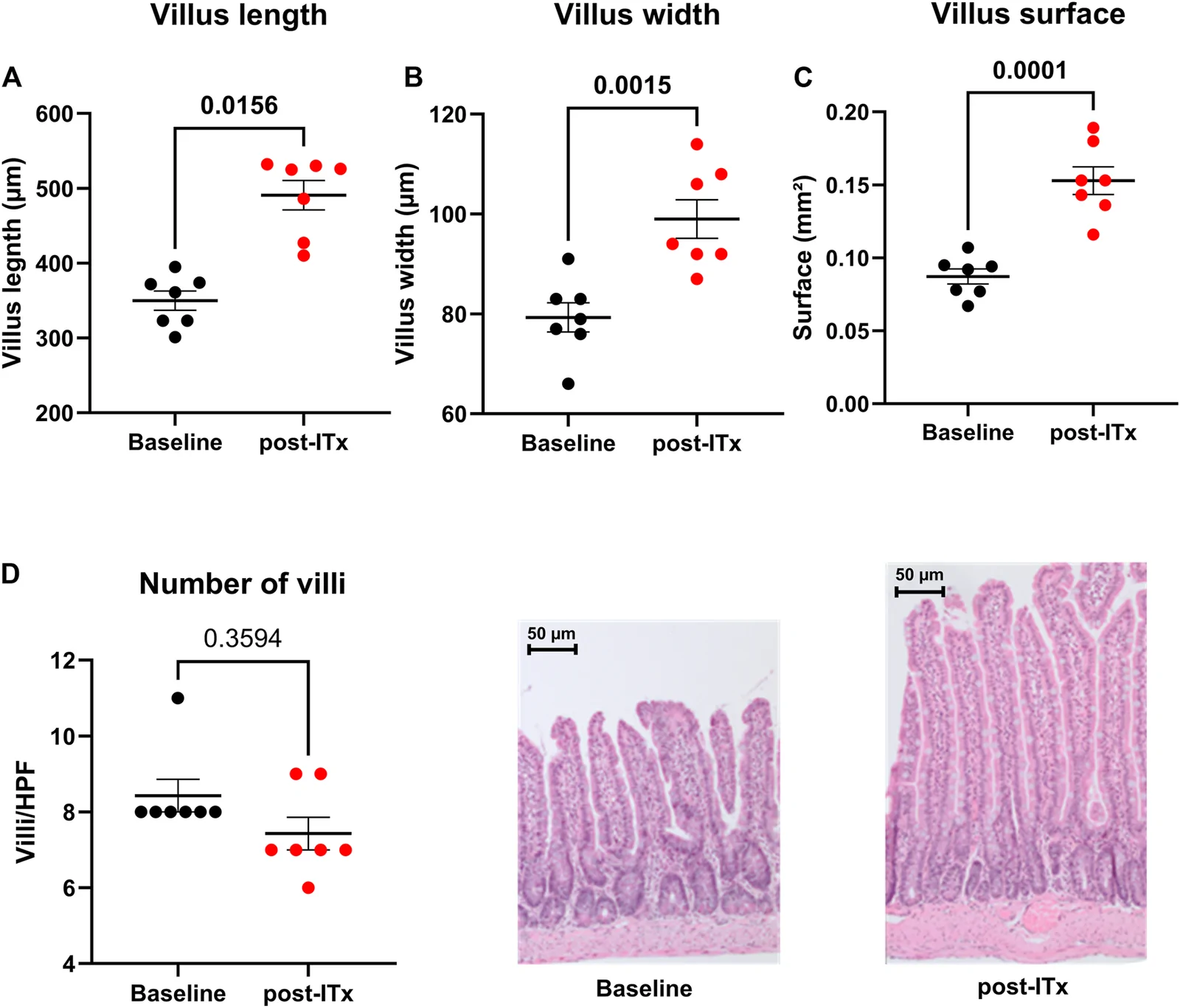
Changes in intestinal anatomy. Histological analysis of pre-procurement (baseline) and post-ITx ileal samples was used to assess absorption determinants (n = 7). Villus length **(A)** and width **(B)**, measured at four locations per sample, were used to calculate villus surface area **(C)** using the formula shown in Figure 2. Villus number **(D)** was determined by counts at two distinct sites. Paired comparisons were performed.

### Reduced Paneth cell number post-ITx without alteration of the crypt/villus ratio

Numbers of crypts and villi per HPF were used to calculate the crypt-to-villus ratio. At baseline, the ratio was 2.0 ± 0.0 crypts per villus per HPF, which remained unchanged post-ITx (2.1 ± 0.1; p > 0.999). In contrast, Paneth cell number was significantly reduced post-ITx compared with baseline (32 ± 4 vs*.* 44 ± 4 cells per HPF; p = 0.030) (Figure 6).

**FIGURE 6:**
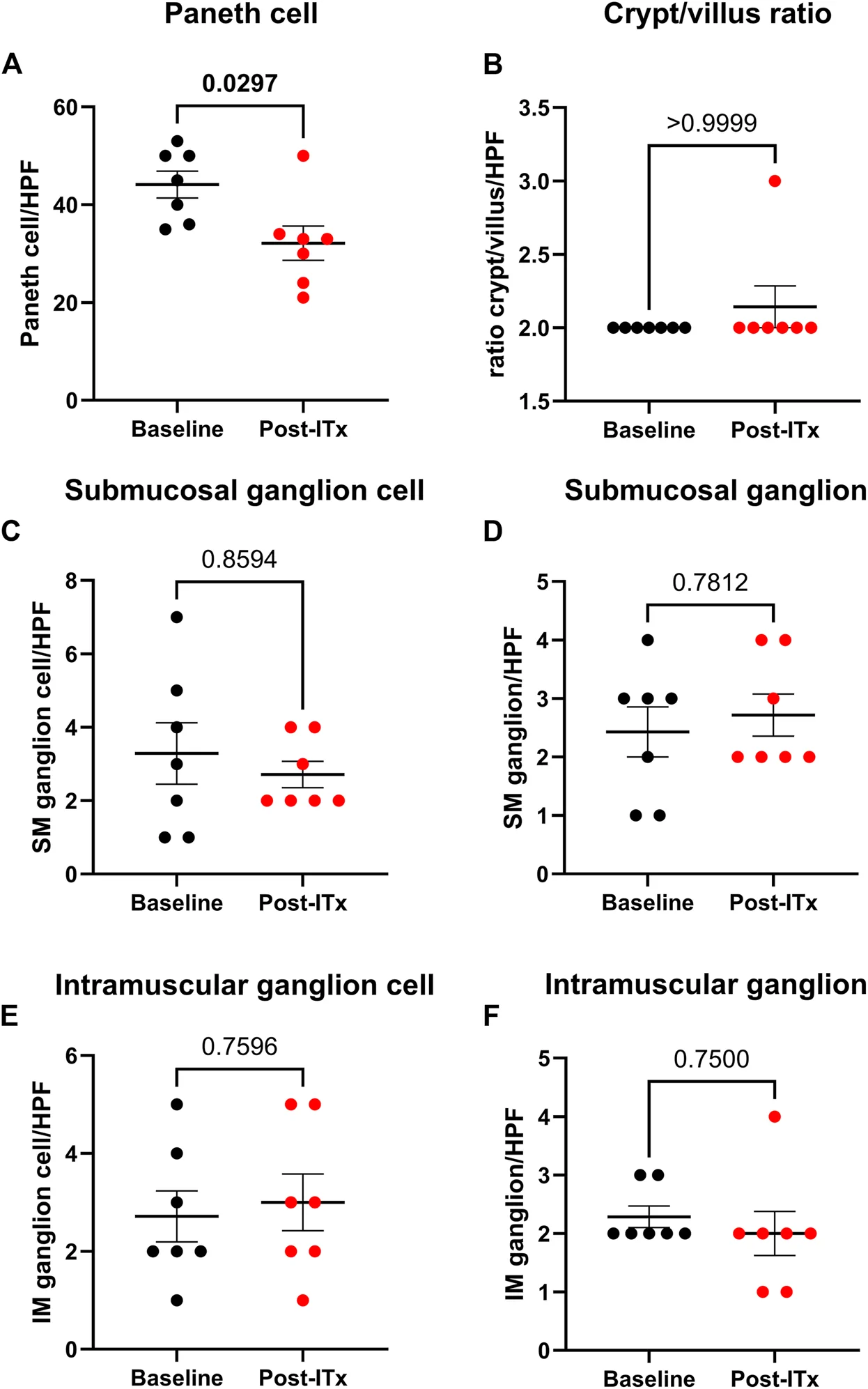
Influence of intestinal transplantation on specific cells. **(A)** Paneth cells were counted per high-power-field, at two locations, and averaged in pre-procurement (Baseline) and post-ITx ileal samples (n = 7). **(B)** Since Paneth cells are primarily located in crypts, the ratio of crypts to villi per high-power field was calculated from counts at two locations per slide. Paired comparisons were performed. **(C,D)** Submucosal ganglia and neurons were counted at two locations, averaged for each animal in both group (Baseline, post-ITx), and compared using paired analysis. **(E,F)** Intermuscular ganglia and neurons were counted and compared using the same approach. HPF, high power field; IM, intramuscular; ITx, intestinal transplantation; SM, submucosal.

### Preserved enteric nervous system despite post-ITx graft denervation

In the submucosal plexus, the number of ganglia per HPF was similar at baseline and post-ITx (2.43 ± 0.43 vs*.* 2.71 ± 0.36; p = 0.781), while the number of neurons per ganglion was also comparable (3.29 ± 0.84 vs*.* 2.71 ± 0.36; p = 0.859). Likewise, the ganglia were preserved in the myenteric plexus, with no difference in ganglion number (2.27 ± 0.18 vs*.* 2.00 ± 0.38 ganglia per HPF; p = 0.750) or in the number of neurons per ganglion (2.71 ± 0.52 vs. 3.00 ± 0.58; p = 0.760) (Figure 6).

## Discussion

In this pilot study, we investigated intestinal graft permeability and motility 2 days before and 4 weeks after syngeneic ITx using orally administered fluorescent dextran. We observed a significant increase in paracellular absorption after ITx, whereas transcellular absorption was unchanged. This increase may be partly explained by an increased post-transplant VL and VW, which expands the absorptive surface area. Interestingly, these functional and morphological changes align with previous findings reported in the literature, using the same model [17, 18].

Molecular weight is a primary determinant of intestinal permeability, as demonstrated in Ussing chamber studies [19]. The paracellular pathway, regulated by tight-junction integrity, mainly mediates the transport of ions, water, and hydrophilic molecules with molecular weights between approximately 0.4 and 20 kDa [11]. In contrast, the transcellular pathway comprises three major mechanisms: (i) passive diffusion of small hydrophilic and lipophilic compounds (<0.4 kDa), (ii) energy-dependent active transport via specific transporters (e.g., sugars, amino acids, and vitamins), and (iii) endocytosis of larger peptides and proteins, which is the dominant route for high–molecular weight molecules (40–80 kDa) [11]. Fluorescent dextrans are validated tools for assessing intestinal permeability and enable discrimination between these pathways based on molecular weight [8]. Accordingly, we used 4 kDa dextran to evaluate paracellular permeability and 70 kDa dextran to assess transcellular transport, the latter being more selective for endocytosis [19, 20].

The timing of sampling helps to identify the intestinal site of dextran absorption. Based on GI transit kinetics, circulating dextran levels reflect sequential uptake from the jejunum, ileum, and colon [21, 22]. In rats, small intestinal transit occurs within approximately 2–6 h after gavage, with the 6-h time point already including partial colonic exposure [23]. In our model, post-ITx overall GI transit time was similar compare to Baseline, although we did not differentiate between small intestine and colonic transit.

Our results demonstrate an increased paracellular permeability after ITx, whereas transcellular permeability was unchanged and even reduced at 24 h. Because paracellular transport is driven by concentration gradients and tight-junction integrity, increased 4 kDa dextran uptake may primarily reflect structural alterations of the inter-epithelial compartment. The transcellular pathway, which represents a much larger absorptive surface, depends on epithelial metabolism and seems preserved in our experiment [24, 25]. We also observed increased VL and VW, indicating an expansion of the absorptive surface. This may also explain the heightened paracellular flux, whereas transcellular permeability remains self-limited by slower, energy-dependent transepithelial transport [26]. Increased occludin expression post-ITx appears to support this second hypothesis, although insufficient to discriminate between the two.

A global increase in absorption surface area would be expected to enhance both FITC4 and TRITC70 permeability. However, only paracellular permeability was increased. One potential explanation may be the graft hypercontractility. Accelerated intestinal transit could reduce mucosal contact time, thereby limiting absorption [25]. This effect may be more pronounced for transcellular than for passive paracellular transport, consistent with the findings of Yamamoto et al. They showed that small intestine absorption in Wistar rats decreases with increasing molecular weight, an effect that may be further exacerbated by accelerated transit. [27]. The subsequent increased delivery of dextran to the colon is unlikely to significantly affect plasma levels, given its poor colonic permeability, as suggested by PEG studies showing minimal colonic absorption of molecules ≥0.6 kDa [28]. Furthermore, accelerated small intestine transit may also contribute to the observed mucosal adaptations by functionally mimicking reduced small bowel length. This could stimulate compensatory mucosal remodeling, including increased VL and VW. Such adaptations have been described after ITx, although they may also reflect ongoing epithelial regeneration triggered by IRI [20, 29].

Graft hypercontractility have been suggested by several reports in literature. *Ex vivo* electrical stimulation studies in syngeneic ITx models demonstrate increased jejunal contractility with a shift in neurotransmission within the first 4 weeks after transplantation, from hypercholinergic to non-adrenergic-non-cholinergic pathways [30, 31]. In contrast, studies evaluating the impact of IRI on interstitial cells of Cajal report no change in the strength or frequency of smooth-muscle contraction [3]. Finally, Hirose et al. observed preserved neuronal, glial, and peptidergic markers after isogeneic jejunal transplantation, with evidence of partial re-innervation up to 400 days post-ITx [5]. Our results support these findings, showing no differences in intramuscular and submucosal ganglia and ganglion cells, although graft-specific transit time could not be assessed due to methodological limitations. Together, these data highlight the complexity of graft motility regulation after ITx, which complicates its pharmacological control despite preserved anatomical structure [30, 32].

Intestinal regeneration following injury is initiated by intestinal stem cells located at the base of the crypts. These cells differentiate into absorptive progenitors (*e.g.*, enterocytes) or secretory lineages, including goblet cells, enteroendocrine cells, and Paneth cells [33]. Among the pathways regulating regeneration, Paneth cells play a central role by supporting and stimulating intestinal stem cell activity. Located within the crypts, they contribute to stem cell maintenance, modulation of the microbiota, phagocytosis, heavy metal handling, and preservation of epithelial barrier integrity through the secretion of antimicrobial peptides [34]. In humans, Paneth cell depletion has been reported in several inflammatory intestinal conditions, including celiac disease, active Crohn’s disease, necrotizing enterocolitis, and graft-versus-host disease [34–36]. Similarly, IRI is associated with reduced Paneth cell numbers, as demonstrated by Grootjans et al [37]. In the context of ITx, Kip et al. reported an early post-transplant decrease in Paneth cell numbers, although no direct association with IRI was established as Paneth cell apoptosis was not observed immediately after reperfusion [38]. Our findings are consistent with these observations and suggest that reduced Paneth cell numbers persist at 4 weeks post-transplantation.

This study has several limitations. First, the limited sample size may have precluded the detection of small differences in the studied parameters. Second, a 4-week follow-up may not adequately reflect long-term graft adaptation [39]. Reinnervation of the intestinal graft has been reported up to 400 days after ITx, and lymphatic reconnection typically occurs within 4–10 weeks post-transplantation [30]. Nevertheless, intestinal function appeared recovered, as indicated by normal recipient weight gain at 4 weeks in most rat ITx protocols [40]. In addition, normal long-chain triglyceride absorption, primarily mediated by lymphatic vessels, has been reported as early as 15 days after transplantation [41]. Third, intestinal permeability was assessed according to dextran molecular weight. Although this approach provides insight into graft adaptation, it does not fully predict nutrient or drug absorption, which primarily occurs via transcellular pathways and depends on multiple additional factors, thereby limiting the translational applicability of our findings [24]. Forth, despite known strain-dependent variability, overall GI transit was faster than previously reported [23, 42]. A more detailed assessment of motility would have required electrophysiological studies or graft-specific tracers. Fifth, a deeper analysis of intestinal barrier integrity and tight junctions may help determine whether changes in permeability reflect impaired barrier function or increased absorption surface. Moreover, the inclusion of a sham group would help distinguish these changes from those induced by the surgical stress. Finally, our experimental design did not account for the impact of immunosuppression on post-transplant intestinal adaptation, despite its central role in clinical ITx and its potential influence on graft physiology.

## Conclusion

This pilot study provides novel insights into intestinal isograft physiology and histology. Using fluorescent dextran, we observed increased paracellular permeability with preserved transcellular absorption 4 weeks after ITx. Concurrently, villus height and width were increased, resulting in expansion of the absorptive surface. Overall GI motility was unchanged post-ITx, although small intestinal and colonic transit were not differentiated. In addition, this study demonstrates the feasibility of using fluorescent dextran to evaluate *in vivo* intestinal permeability and global transit. Further research is required to assess long-term adaptations beyond 4 weeks post-transplant and the impact of immunosuppression on graft function. Additionally, more comprehensive assessments of intestinal barrier integrity and electrophysiology may help elucidate the mechanisms underlying the observed changes.

## Data Availability

The raw data supporting the conclusions of this article will be made available by the authors, without undue reservation.
